# Molecular analysis of clinical isolates previously diagnosed as *Mycobacterium intracellulare* reveals incidental findings of “*Mycobacterium indicus pranii*” genotypes in human lung infection

**DOI:** 10.1186/s12879-015-1140-4

**Published:** 2015-09-30

**Authors:** Su-Young Kim, Hye Yun Park, Byeong-Ho Jeong, Kyeongman Jeon, Hee Jae Huh, Chang-Seok Ki, Nam Yong Lee, Seung-Jung Han, Sung Jae Shin, Won-Jung Koh

**Affiliations:** Division of Pulmonary and Critical Care Medicine, Department of Medicine, Samsung Medical Center, Sungkyunkwan University School of Medicine, Seoul, South Korea; Department of Laboratory Medicine and Genetics, Samsung Medical Center, Sungkyunkwan University School of Medicine, Seoul, South Korea; Department of Microbiology, Institute for Immunology and Immunological Diseases, Brain Korea 21 PLUS Project for Medical Science, Yonsei University College of Medicine, Seoul, South Korea

**Keywords:** *Mycobacterium intracellulare*, *Mycobacterium indicus pranii*, Multigene sequence-based typing, Insertion elements

## Abstract

**Background:**

*Mycobacterium intracellulare* is a major cause of *Mycobacterium avium* complex lung disease in many countries. Molecular studies have revealed several new Mycobacteria species that are closely related to *M. intracellulare*. The aim of this study was to re-identify and characterize clinical isolates from patients previously diagnosed with *M. intracellulare* lung disease at the molecular level.

**Methods:**

Mycobacterial isolates from 77 patients, initially diagnosed with *M. intracellulare* lung disease were re-analyzed by multi-locus sequencing and pattern of insertion sequences.

**Results:**

Among the 77 isolates, 74 (96 %) isolates were designated as *M. intracellulare* based on multigene sequence-based analysis. Interestingly, the three remaining strains (4 %) were re-identified as “*Mycobacterium indicus pranii*” according to distinct molecular phylogenetic positions in *rpoB* and *hsp65* sequence-based typing. In *hsp65* sequevar analysis, code 13 was found in the majority of cases and three unreported codes were identified. In 16S–23S rRNA internal transcribed spacer (ITS) sequevar analysis, all isolates of both species were classified within the Min-A ITS sequevar. Interestingly, four of the *M. intracellulare* isolates harbored IS*1311*, a *M. avium*-specific element. Two of three patients infected with “*M. indicus pranii*” had persistent positive sputum cultures after antibiotic therapy, indicating the clinical relevance of this study.

**Conclusions:**

This analysis highlights the importance of precise identification of clinical isolates genetically close to *Mycobacterium* species, and suggests that greater attention should be paid to nontuberculous mycobacteria lung disease caused by “*M. indicus pranii*”.

## Background

*Mycobacterium avium* complex (MAC) lung disease is the most common lung disease caused by nontuberculous mycobacteria (NTM) and its prevalence has been increasing worldwide [1–10]. MAC was originally composed of two species, *M. avium* and *Mycobacterium intracellulare* [11]. Many laboratories and studies reported these species as MAC because they are highly similar, and the clinical features of patients who are infected with these two species are considered indistinguishable [11, 12]. However, some studies have suggested that differentiation between *M. avium* and *M. intracellulare* may have epidemiologic and clinical relevance [13, 14].

*M. intracellulare* is a major cause of MAC lung disease in many countries [2]. Molecular studies have revealed the presence of additional taxonomic groups or sequence variants (sequevars) that are closely related to *M. intracellulare.* Several new species were recently identified including *Mycobacterium chimaera*, *Mycobacterium colombiense*, *Mycobacterium arosiense*, *Mycobacterium vulneris*, *Mycobacterium marseillense*, *Mycobacterium timonense*, *Mycobacterium bouchedurhonense*, *Mycobacterium mantenii*, and *Mycobacterium yongonense* [15–20]. However, data regarding the proportion of these new species that are etiologic organisms in patients with previously diagnosed *M. intracellulare* lung disease are very limited.

The methods of correct identification for mycobacterial species in clinical laboratories have changed dramatically over the past two decades. Molecular methods have now surpassed biochemical tests and high-performance liquid chromatography as the method of choice for identifying NTM [21]. Polymerase chain reaction (PCR) restriction fragment length polymorphism analysis (PRA) based on restriction digestion products of specific genes such as *hsp65*, 16S rRNA, *rpoB*, and 16S–23S rRNA internal transcribed spacer (ITS) has been reported as a rapid, feasible, and inexpensive diagnostic method [22–25]. The gold standard identification method of 16S rRNA gene sequencing and sequencing of each loci as a single identification target has failed to discriminate closely related *Mycobacterium* species such as MAC, *Mycobacterium abscessus*-*Mycobacterium chelonae*, *Mycobacterium farcinogenes*-*Mycobacterium senegalense*, *Mycobacterium kansasii*-*Mycobacterium gastri*, and *Mycobacterium marinum*-*Mycobacterium ulcerans* [26, 27]. Recently, multigene sequence-based typing has been suggested as the new standard method for identifying *Mycobacterium* species that are not well discriminated by 16S rRNA gene sequences alone [8, 28–30].

In our institution, the *rpoB*-PRA method had been used for species identification and diagnosis of MAC lung disease [14]. Recently published papers have emphasized the importance of taxonomy in distinguishing the many species and subspecies of MAC. Nonsequencing methods or 16S rRNA sequencing might fail to distinguish closely related species [31, 32], indicating that nonsequencing-based approaches or analysis of a single target are not suitable for the accurate identification of (sub-)species belonging to MAC.

Thus, the aim of this study was to re-identify clinical isolates from patients previously diagnosed with *M. intracellulare* lung disease and to characterize their molecular pattern. For this purpose, the following methods were used: (1) multigene sequence-based typing of 16S rRNA, *rpoB*, *hsp65* and ITS genes, (2) *hsp65* and ITS sequevar-based classification, and (3) insertion element analysis. Finally, three “*Mycobacterium indicus pranii*” strains that were previously identified as *M. intracellulare* were re-identified. The clinical characterization of lung disease caused by these three “*M. indicus pranii*” infections was described. “*M. indicus pranii*” is of specific interest due to its evolutionary significance and therapeutic potential in various disease processes. This study raises the possibility of “*M. indicus pranii*” as a pathogenic organism in the appropriate host and clinical situation, a notion not previously suggested in prior publications.

## Methods

### Study subjects

Clinical isolates from 77 consecutive patients who were newly diagnosed with *M. intracellulare* lung disease from Jan. 2008 to Dec. 2009 at Samsung Medical Center (a 1,961-bed referral hospital in Seoul, Korea) were collected and stored. This study was approved by the Institutional Review Board of Samsung Medical Center (File No. 2008-09-016). All patients' record and information was anonymized and de-identified prior to analysis. All patients met the diagnostic criteria for NTM lung disease [11]. All patients were immunocompetent and none of the patients tested positive for human immunodeficiency virus. Baseline patient characteristics are summarized in Table 1.

**Table 1:** Clinical characteristics of 77 patients with previously diagnosed *M. intracellulare* lung disease

	No. (%) or median (IQR)
Age, years	64 (55–72)
Female	40 (52)
Body mass index (kg/m^2^)	20.0 (17.7–21.5)
Non-smoker	55 (71)
Previous history of TB treatment	43 (56)
Positive sputum AFB smear	39 (51)
Type	
Nodular bronchiectatic form	47 (61)
Fibrocavitary form	22 (29)
Unclassifiable form	8 (10)

*IQR* interquartile ranges, *TB* tuberculosis, *AFB* acid-fast bacilli

The isolates were collected before initiating antibiotic treatment for *M. intracellulare* lung disease. NTM species were identified as *M. intracellulare* by PRA based on the *rpoB* gene at time of diagnosis [14].

### Identification of patient isolates by multigene sequence-based typing

NTM were propagated in Middlebrook 7H9 broth (Difco Laboratories, Detroit, MI, USA) supplemented with 10 % (vol/vol) oleic acid-albumin-dextrose-catalase (OADC; BD Diagnostics). Mycobacterial DNA was extracted using a DNeasy Blood and Tissue Kit according to the manufacturer’s instructions (Qiagen, Valencia, CA). Multigene sequence-based typing including *hsp65*, *rpoB*, ITS and 16S rRNA fragments was carried out using PCR primer sets as described previously (Table 2). The PCR products of target genes were subjected to sequence analysis. *hsp65* and ITS sequevar analysis were performed as previously described [33, 34]. The nucleotide sequences of these genes were compared with data reported by BLAST analysis (http://www.ncbi.nlm.nih.gov/) against sequences from *M. intracellulare* ATCC13950^T^, *M. intracellulare* ATCC15985, *M. intracellulare* MOTT-64, *M. intracellulare* MOTT-02, *M. intracellulare* MOTT36Y, and “*M. indicus pranii*” MTCC9506. For phylogenetic analysis, sequences were trimmed using the CLUSTAL-W multiple sequence alignment program [35]. Phylogenetic trees were obtained from DNA sequences utilizing the neighbor-joining method and Kimura’s two parameter distance correction model with 1000 bootstrap replications supported by MEGA 6.0 software [36].

**Table 2:** Primers used in this study

Target	Sequence (5′ to 3′) of paired primers	Reference
16S rRNA	AGA GTT TGA TCC TGG CTC AG	[54]
	GTA TTA CCG CGG CTG CTG	
ITS	TTG TAC ACA CCG CCC GTC	[34]
	TCT CGA TGC CAA GGC ATC	
*hsp65*	AAC GTC GTC CTG GAG AAG AA	[55]
	GCC TTC TCC GGC TTG TC	
*rpoB*	GGC AAG GTC ACC CCG AAG GG	[27]
	AGC GGC TGC TGG GTG ATC ATC	
IS*900*	TGG ACA ATG ACG GTT ACG GAG GTG G	[37]
	CGC AGA GGC TGC AAG TCG TGG	
IS*901*	CGA CGA CAG GAG TAG CGG TAT GGC	[38]
	CCG TGC TGC GAG TTG CTT GAT GAG	
IS*1311*	GCG TGA GGC TCT GTG GTG AA	[37]
	ATG ACG ACC GCT TGG GAG AC	
DT1	CGT TGG CTG GCC ATT CAC GAA GGA GT	[37]
	GCT AGT TGG ATC GCG CCG AAC ACC GG	

### Insertion element analysis

Multiplex PCR was performed to detect four target genes, IS*900*, IS*901*, IS*1311* and DT1, using previously described methods [37, 38]. PCR product sizes of 398 bp, 754 bp, 608 bp, and 296 bp corresponded to amplification of IS*900*, IS*901*, IS*1311*, and DT1 targets, respectively (data not shown). Amplification of only the DT1 gene indicated *M. intracellulare*. PCR products of insertion elements were sequenced and the existence of a specific insertion element in each strain was confirmed. DNA isolated from *Mycobacterium abscessus* ATCC19977, *Mycobacterium tuberculosis* H37Rv ATCC27294, and *Mycobacterium gastri* ATCC15754 were used as negative controls for each primer set in each PCR run.

## Results

### Re-identification of clinical isolates by multigene sequence-based typing

**Fig. 1:**
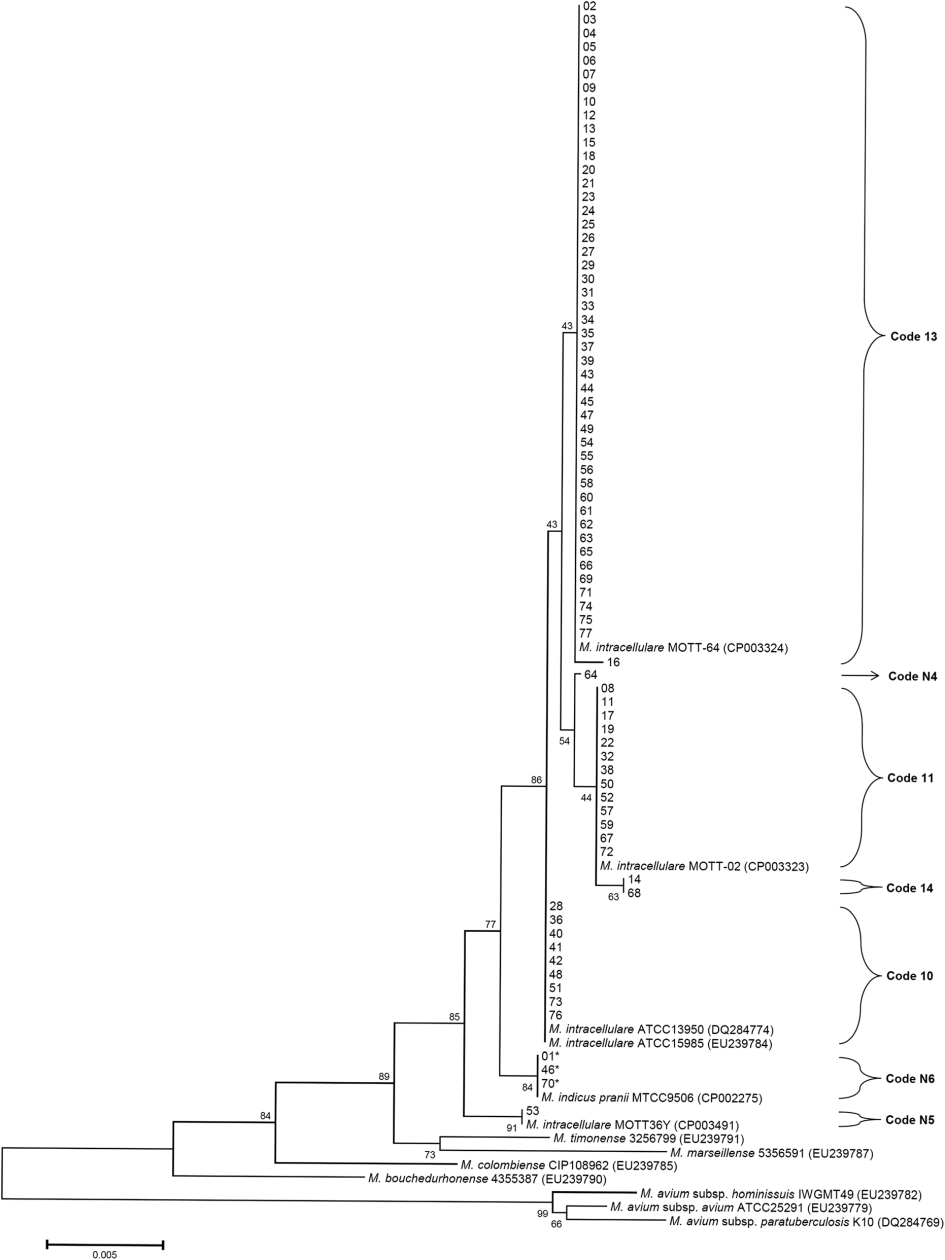
A *hsp65* sequence-based phylogenetic tree of 77 isolates including the *M. intracellulare* type, *M. intracellulare* clinical strains, “*M. indicus pranii*”, and other MAC species using the neighbor-joining method with Kimura’s two parameter distance correction model. Bootstrap analyses determined from 1000 replicates are indicated at the nodes. Bar, 0. 5 % difference in nucleotide sequence. GenBank accession numbers are given in parentheses

**Fig. 2:**
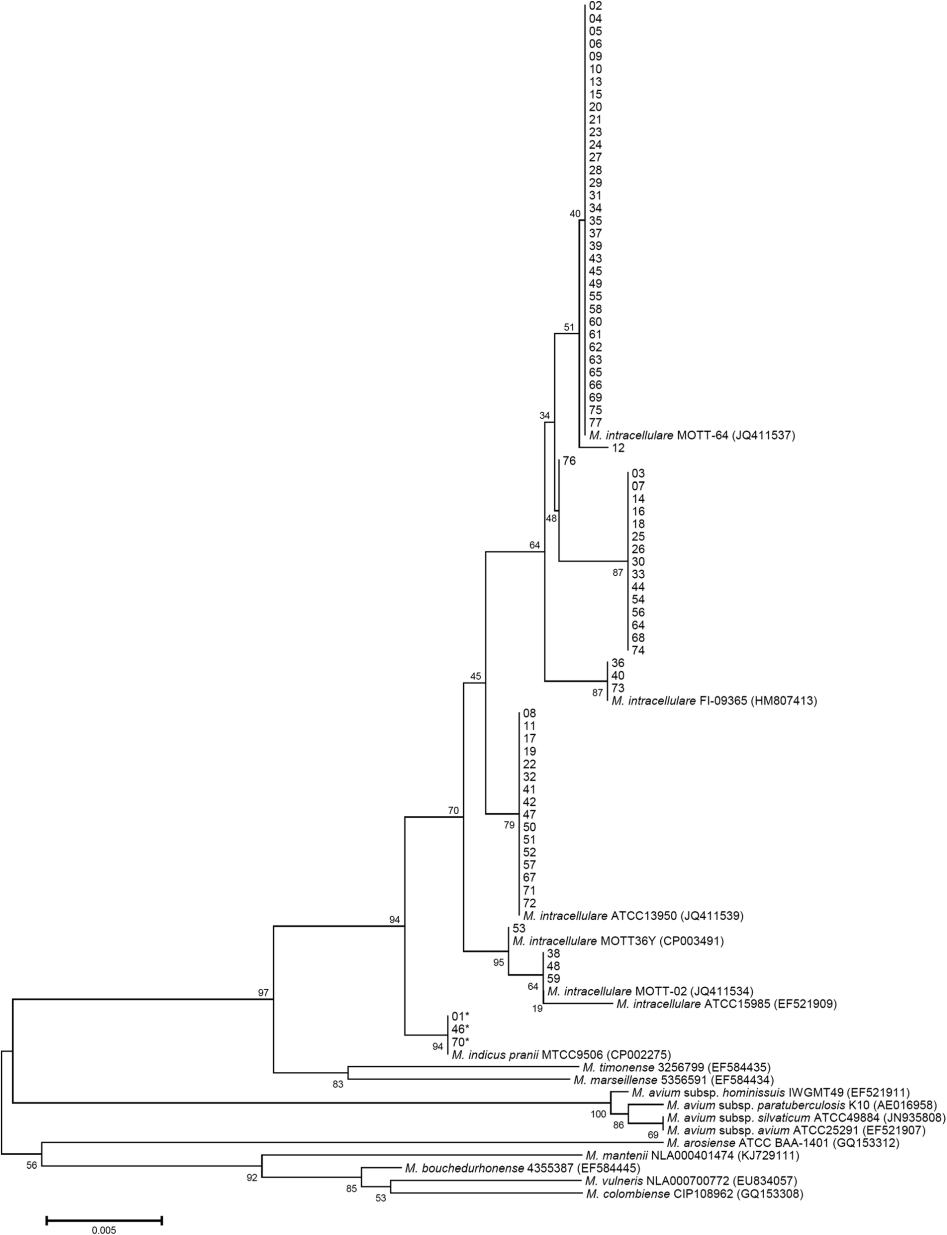
The *rpoB* sequence-based phylogenetic tree of 77 isolates including the *M. intracellulare* type, *M. intracellulare* clinical strains, “*M. indicus pranii*”, and other MAC (sub-)species using the neighbor-joining method with Kimura’s two parameter distance correction model. Bootstrap analyses determined from 1000 replicates are indicated at the nodes. Bar, 0.5 % difference in nucleotide sequence. GenBank accession numbers are given in parentheses

**Fig. 3:**
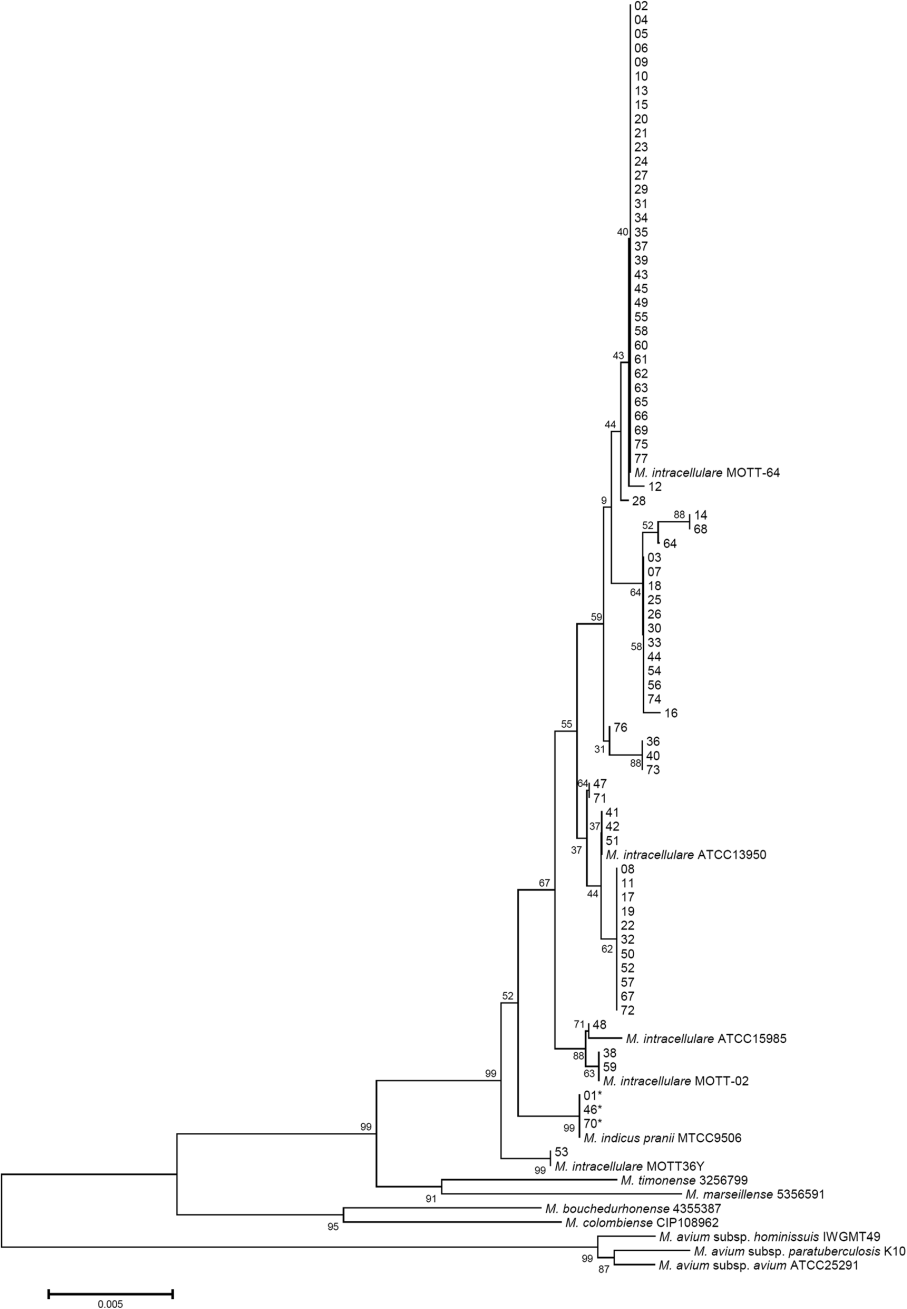
The phylogenetic tree based on concatenated *hsp65* and *rpoB* sequences of 77 isolates including *M. intracellulare* type, *M. intracellulare* clinical strains, “*M. indicus pranii*”, and other MAC (sub-)species using the neighbor-joining method with Kimura’s two-parameter distance correction model. Bootstrap analyses determined from 1000 replicates are indicated at the nodes. Bar, 0.5 % difference in nucleotide sequence. GenBank accession numbers are shown in Figs. 1 and 2

Isolates from 77 patients diagnosed with *M. intracellulare* lung disease were re-identified. Clinical isolates from 74 (96 %) patients were identified as *M. intracellulare* and those from three (4 %) patients were identified as “*M. indicus pranii*” using multiple gene sequencing analysis (Table 3). The 16S rRNA and ITS sequences of “*M. indicus pranii*” isolates were identical to those of the “*M. indicus pranii*” type strain (GenBank accession no. CP002275) and the *M. intracellulare* type strain (GenBank accession nos. GQ153276 and CP003322, respectively). However, the *rpoB* and nearly complete *hsp65* sequences (PCR with *hsp65*-sequevar primer sets) of “*M. indicus pranii*” isolates (isolate 01, 46 and 70) were only identical to those of the “*M. indicus pranii*” type strain (GenBank accession no. CP002275). They were 99.6 % (708/711) and 99.8 % (1413/1416) similar to the *rpoB* and *hsp65* sequences of the *M. intracellulare* type strain (GenBank accession nos. JQ411539 and DQ284774, respectively). The phylogenetic tree of all isolates with *M. intracellulare* and “*M. indicus pranii*” type strains is shown in Figs. 1, 2 and 3.

**Table 3:** Re-identification using multigene sequence-based typing, distribution of *hsp65*, ITS sequevar analysis, and insertion elements

	*M. intracellulare* (MI)	“*M. indicus pranii*” (MIP)	Comparison between MI and MIP
Identification and diagnosis by			
Non-sequencing method (PRA)	77	0	Identical
Multigene sequence-based typing	74	3	Different
16S rRNA			Identical
ITS			Identical
*rpoB*			Different
*hsp65*			Different
Molecular characterization			
Distribution of *hsp65* sequevar			Different^a^
Code 10	9	0	
Code 11	13	0	
Code 13	48	0	
Code 14	2	0	
Code N4	1	0	
Code N5	1	0	
Code N6	0	3	
ITS sequevar	Min-A	Min-A	Identical
Insertion elements^b^			Identical
IS*900*	-	-	
IS*901*	-	-	
IS*1311*	−/+^c^	-	
DT1	+	+	

*PRA*, PCR restriction fragment length polymorphism analysis, *ITS* internal transcribed spacer

^a^Two species were not distinguished by previously published *hsp65* code, but code N6 identified in this study was different between the two species

^b^PCR results of insertion element are indicated as positive(+) or negative(−)

^c^Four isolates identified as *M. intracellulare* were positive for IS*1311*

In all, the 77 isolates were classified to seven different *hsp65* sequevars according to the method described by Turenne *et al.* [33]. Four of these sequevars were well recognized as *M. intracellulare* type and related strains, and 3 were newly identified in this study. The new sequevars were coded N4, N5 and N6 [followed by the code name given in the previous paper [39]. Ten single nucleotide polymorphisms (SNPs) excluding SNPs reported in the previous study were identified in this study (Table 4). The distribution of *hsp65* sequevars in the 77 isolates is shown in Tables 3 and 4. In all, 74 *M. intracellulare* and three “*M. indicus pranii*” isolates were classified as the Min-A ITS sequevar.

**Table 4:** Identification of novel *hsp65* sequevar codes and *hsp65* SNPs among “*M. indicus pranii*” and *M. intracellulare* clinical strains compared to the *M. intracellulare* type strain

*hsp65* code^a^	Species or strain	Nucleotide at the indicated base pair position (*hsp65*)^b^																		No. of isolates
		192	**198**	249	279	285	459	**477**	**555**	**633**	**726**	**804**	**921**	**933**	**1011**	**1191**	1371	1423	1467	
Code 10	*M. intracellulare* ATCC13950	G	G	C	G	T	C	C	C	G	C	C	C	T	G	G	G	C	C	9
Code 11	*M. intracellulare* FCC1804	•	•	•	•	•	•	•	•	•	•	•	•	•	•	•	•	•	T	13
Code 12	*M. intracellulare* 96006	•	•	•	•	•	T	•	•	•	•	•	•	•	•	•	•	•	•	0
Code 13	*M. chimaera* MI-JC	T	•	T	T	C	•	•	•	•	•	•	•	•	•	•	•	T	•	48
Code 14	*M. intracellulare* 90331	•	•	•	•	•	•	•	•	•	•	•	•	•	•	•	A	•	T	2
Code N4^c^	*M. intracellulare* clinical isolate 64	T	A	T	T	C	•	•	•	•	•	•	•	•	•	•	•	T	T	1
Code N5^c^	*M. intracellulare* MOTT36Y	•	A	•	•	•	•	G	G	C	T	•	G	C	C	C	•	•	•	1
Code N6^c^	“*M. indicus pranii*” MTCC9506	•	•	•	•	•	•	•	•	•	•	G	•	•	C	C	•	•	•	3

^a^Classification according to Turenne et al. [33]

^b^ • indicates the same base pair as in code 10; New base pair position found in this study are indicated by bold font

^c^New code types found in this study are designated by code N4, N5, and N6

### Distribution of insertion elements between *M. intracellulare* and *M. indicus pranii* strains

All isolates were negative for IS*900* (considered diagnostic for *M. avium* subsp. *paratuberculosis*) as well as IS*901* (considered diagnostic for *M. avium* subsp. *avium*), and positive for DT1 (considered diagnostic for *M. intracellulare* and *M. avium* subsp. *avium*). Interestingly, four (5 %) isolates identified as *M. intracellulare* were positive for IS*1311* (considered diagnostic for all members of *M. avium* subspecies). The IS*1311* sequences of four *M. intracellulare* isolates were identical to those of the *M. avium* insertion sequence IS*1311* transposase gene (GenBank accession no. U16276), indicating that IS*1311* might truly exist in some *M. intracellulare* strains.

### Clinical characteristics of three patients with *M. indicus pranii* lung disease

**Fig. 4:**
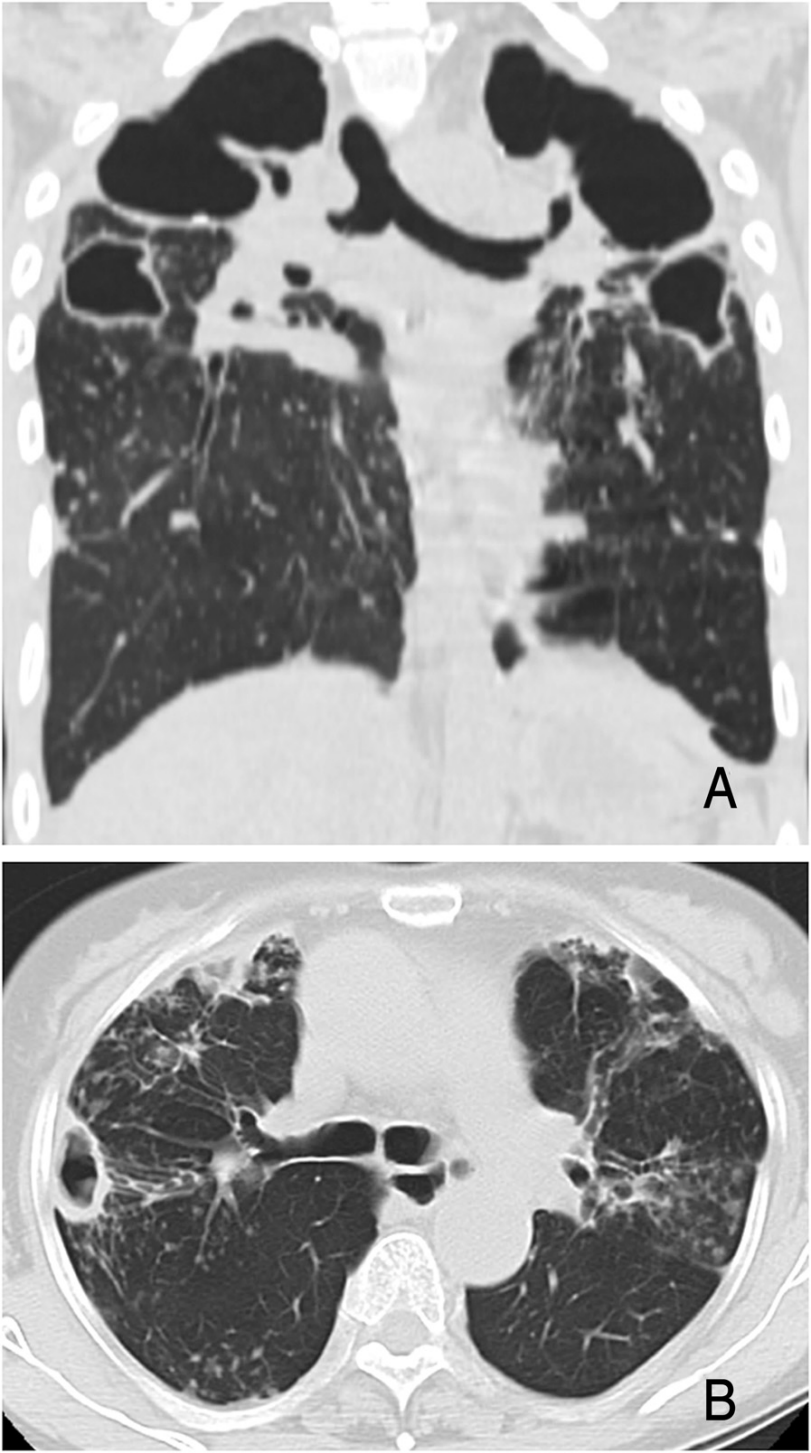
High-resolution computed tomography (HRCT) findings of “*M. indicus pranii*” lung disease. **a** A 27-year-old male with a prior history of pulmonary tuberculosis. Chest HRCT shows multiple bilateral large cavities in both upper lobes. The patient died after initiation of combination antibiotic therapy due to an accident. **b** A 72-year-old female. Chest HRCT shows severe bronchiectasis in the right middle lobe and lingular segment of the left upper lobe. Note a cavitary lesion in the right middle lobe, as well as multiple small nodules and tree-in-bud appearance in both lungs

Three patients were re-diagnosed as having “*M. indicus pranii*” lung disease (Table 5 and Fig. 4). Two patients received combination antibiotic therapy including clarithromycin, ethambutol, rifampin, and streptomycin. Three isolates from each patient were identified as “*M. indicus pranii*” using multigene sequence-based typing and had no mutations in *rrl* (23S rRNA gene) according to sequencing analysis, which is known as main mechanism of acquired macrolide resistance in MAC [40]. Patient 1 died of an accident after five months of antibiotic therapy, and patient 2 showed persistent positive sputum cultures after 24 months of antibiotic therapy. Patient 3 was followed up without antibiotic treatment for 5.5 years because of mild symptoms.

**Table 5:** Clinical characteristics of three patients with “*M. indicus pranii*” lung disease

	Patient 1	Patient 2	Patient 3
Sex/Age	M/27	F/72	M/42
Previous TB treatment	Yes	No	No
Sputum AFB smear	Positive	Positive	Negative
Radiographic type	Fibrocavitary	Nodular bronchiectatic	Nodular bronchiectatic
Cavitary lesion	Bilateral	Unilateral	None
Clarithromycin MIC (μg/mL)	1.0	1.0	≤0.5
Antibiotic treatment	Yes	Yes	No
Treatment outcomes	Death after 5 months of treatment	Persistent positive sputum culture after 24 months of treatment	Follow-up without treatment

*TB* tuberculosis, *AFB* acid-fast bacilli, *MIC* minimum inhibitory concentration

## Discussion and conclusions

In this study, clinical isolates from 77 patients that were previously diagnosed with *M. intracellulare* lung disease over a two-year period were re-identified. Species identification was initially performed by a non-sequencing method and then species were re-identified using a sequencing method. Among the 77 isolates identified as *M. intracellulare* by PRA at the time of diagnosis, 74 isolates were repeatedly identified as *M. intracellulare.* The remaining three were re-identified as “*M. indicus pranii*” by multigene sequence-based typing. However, *hsp65* and ITS sequevar analyses were not precise enough to discriminate between *M. intracellulare* and “*M. indicus pranii*” in this study. To our knowledge, this is the first study to report documented cases of “*M. indicus pranii*” lung disease in humans.

*M. intracellulare* isolate 64 with code N4 and isolate 53 with code N5 were negative for IS*900*, IS*901,* and IS*1311*, and positive for DT1. Since classification among MAC subsets based on the *hsp65* sequevar has been proposed [33], there have been several studies published on *M. avium hsp65* sequevars, but none on *M. intracellulare hsp65* sequevars. The distribution of *M. intracellulare hsp65* sequevars in other countries is unknown. However, two-thirds of the strains from this Korean-based study were code 13 type, indicating that further studies to characterize this species are needed.

In general, IS*1311* is present in all members of the *M. avium* subspecies and is not present in *M. intracellulare*. Four *M. intracellulare* isolates possessed identical IS*1311* to that of the *M. avium* in this study, which is a novel observation. Since a number of different IS elements have been described in various NTM species, the species-specific IS elements have been revisited for MAC identification [37, 41, 42]. IS elements are mobile by nature, so there is a risk that similar elements are found in unrelated bacteria because of mobility to or from MAC organisms. Therefore, IS-based PCR differentiation of MAC must be performed in combination with other genetic analyses. Based on this study, DT1 is the optimal candidate marker gene for identification of *M. intracellulare* and “*M. indicus pranii*”*.* Sequences analysis of *hsp65* and *rpoB* provides phylogenetic placement, allowing discrimination between the two species.

“*M. indicus pranii*” is initially named “*Mycobacterium w* (*Mw*)” and used as a potential leprosy vaccine [43]. The use of the name “*Mycobacterium w*” gives an impression that *Mw* is related to the hypervirulent *M. tuberculosis*-W (Beijing strain) strain. To avoid confusion, Talwar *et al.* suggested using the nomenclature “*Mycobacterium indicus pranii*” [44]. However, neither of its name is found on the List of Prokaryotic Names with Standing in Nomenclature, and the designation “*Mycobacterium indicus pranii*” does not conform to the binomial naming convention used for bacterial species [45]. In a recent publication, Alexander *et al.* suggested that “*M. indicus pranii*” is a strain of *M. intracellulare* [46]. “*M. indicus pranii*” is considered to be a non-pathogenic microorganism and no human infections have been reported to date [47]. Use of the “*M. indicus pranii*” vaccine is based on the assumption that antigens shared between *M. tuberculosis* and this saprophytic mycobacterium is relevant for protective immunity and that “*M. indicus pranii*” lacks many of the harmful components present in *M. tuberculosis* [48]. “*M. indicus pranii*” immunotherapy did demonstrate protective efficacy against tuberculosis [49]. However, patients with pericardial tuberculosis who received “*M. indicus pranii*” injections demonstrated no significant benefit with respect to any reported outcomes in recent papers [50, 51]. The efficacy of “*M. indicus pranii*” in severe sepsis has recently been reported [52].

On the basis of our findings, “*M. indicus pranii*” should be considered a cause of pulmonary disease in humans with pre-existing lung disease, such as tuberculosis and bronchiectasis. In addition, the virulence of “*M. indicus pranii*” may vary according to geographical location. “*M. indicus pranii*” could be detected more frequently in the future as a consequence of increased genetic sequencing. Therefore, careful attention should be given to accurately identifying this *Mycobacterium* species. Further studies regarding the pathogenesis of “*M. indicus pranii*”*,* including comparison with *M. intracellulare,* are needed.

Unlike *M. tuberculosis*, which has no environmental reservoir, NTM are ubiquitous microorganisms readily isolated from environmental sources, including soil and water. Despite the reportedly low virulence of NTM in immunocompetent human hosts, an increase in their isolation frequency has been seen in the last decade. Genetic analyses have greatly improved our understanding of the phylogeny and evolutionary diversity of NTM. Our study suggests that precise differentiation of *M. intracellulare* isolates may provide clinically relevant data including ecology, epidemiology, virulence, and treatment outcomes [32, 53].

The precise re-identification of clinical isolates initially identified as *M. intracellulare* by a non-sequencing method in patients with *M. intracellulare* lung disease revealed that most cases were caused by *M. intracellulare*. However, some were caused by “*M. indicus pranii*”. Our study indicates the role of “*M. indicus pranii*” as an agent of severe and chronic lung disease in immunocompetent patients, suggesting that further study is needed to investigate its pathogenicity.

## Abbreviations

MAC: *Mycobacterium avium* complex
NTM: nontuberculous mycobacteria
PCR: polymerase chain reaction
PRA: PCR restriction fragment length polymorphism analysis
ITS: internal transcribed spacer
